# What all students in healthcare training programs should learn to increase health equity: perspectives on postcolonialism and the health of Aboriginal Peoples in Canada

**DOI:** 10.1186/s12909-015-0442-y

**Published:** 2015-09-23

**Authors:** Allana S. W. Beavis, Ala Hojjati, Aly Kassam, Daniel Choudhury, Michelle Fraser, Renee Masching, Stephanie A. Nixon

**Affiliations:** 1 Department of Physical Therapy, University of Toronto, 160-500 University Avenue, Toronto, ON Canada; 2 Global Health Division, Canadian Physiotherapy Association, 955 Green Valley Crescent, Suite 270, Ottawa, ON Canada; 3 International Centre for Disability and Rehabilitation, University of Toronto, 500 University Avenue, Toronto, ON Canada; 4 Canadian Aboriginal AIDS Network, 113-154 Willowdale Drive, Dartmouth, NS Canada

**Keywords:** Postcolonialism, Colonialism, Health equity, Healthcare education, Aboriginal, Cultural safety, Critical consciousness, Curriculum development

## Abstract

**Background:**

The ongoing role of colonialism in producing health inequities is well-known. Postcolonialism is a theoretical approach that enables healthcare providers to better understand and address health inequities in society. While the importance of postcolonialism and health (PCH) in the education of clinicians has been recognized, the literature lacks guidance on how to incorporate PCH into healthcare training programs. This study explores the perspectives of key informants regarding content related to PCH that should be included in Canadian healthcare training programs, and how this content should be delivered.

**Methods:**

This qualitative study involved in-depth, semi-structured interviews with nineteen individuals with insight into PCH in Canada. Data were analyzed collaboratively to identify, code and translate key emergent themes according to the six phases of the DEPICT method.

**Results:**

Three themes emerged related to incorporating PCH into Canadian healthcare training programs: (1) *content* related to PCH that should be taught; (2) *how* this content should be delivered, including teaching strategies, who should teach this content and when content should be taught, and; (3) *why* this content should be taught. For the Canadian context, participants advised that PCH content should include a foundational history of colonization of Aboriginal Peoples in Canada, how structures rooted in colonialism continue to produce health inequities, and how Canadian clinicians’ own experiences of privilege and oppression affect their practice. Participants also advised that this content should be integrated longitudinally through a variety of interactive teaching strategies and developed in collaboration with Aboriginal partners to address health inequities.

**Conclusions:**

These findings reinforce that clinicians and educators must understand health and healthcare as situated in social, political and historical contexts rooted in colonialism. Postcolonialism enables learners to understand and respond to how colonialism creates and sustains health inequities. This empirical study provides educators with guidance regarding PCH content and delivery strategies for healthcare training programs. More broadly, this study joins the chorus of voices calling for critical reflection on the limits and harms of an exclusively Western worldview, and the need for action to name and correct past wrongs in the spirit of reconciliation and justice for all.

## Background

Colonialism has played and continues to play a fundamental role in shaping Canada as nation. Colonization involved the establishment of European settlements in new territories [1], but was not just an historical process [2]. A key feature of colonialism was social stratification through the creation of an “Other” or “the colonized” [1], a process that allowed empires to define themselves as colonizers [1]. Additionally, the concept of “race” emerged during this era as a justification of unequal power relations between settlers and Indigenous peoples [1]. “Culture”, another concept constructed to identify non-European peoples as inferior and uncivilized, was often used in conjunction with race [3]. These strategies were employed historically as tools of colonial conquest and continue to shape the health and illness of people today.

In particular, colonization is an important determinant of health for Indigenous peoples worldwide [2], including in Canada. The *Indian Act,* which is legislation enacted by the Government of Canada in 1876, is now regarded as racist and unjust. For more than a century through this *Act*, the federal government sought to eliminate the “Indian" identity and intensely control the lives of Aboriginal Peoples,^1^ which included the prohibition of traditional healing practices or participation in Aboriginal ceremonies [4, 5]. Under the *Act*, “Indians” are deemed wards of the state [6] and the government dictates who has status as an “Indian” and who does not [7]. The *Indian Act*, placed restrictions on Aboriginal self-government and dispossessed Aboriginal People from their original lands, resulting in severely restricted opportunities to live well in Canadian society [4, 5, 8]. Aboriginal children were forcibly removed from their families and placed in residential schools in order to eliminate their parents’ involvement in their intellectual, cultural and spiritual development [9] – a process now viewed as “cultural genocide” [10]. Despite amendments such as the *First Nations Governance Act*, the *Indian Act* remains the governing policy for and perpetuates the disempowerment of Aboriginal Peoples in Canada today [4, 5]. The last residential school in Canada was only closed in 1996.

The health of Aboriginal Peoples in Canada is powerfully shaped by the structures and attitudes created throughout this history. Aboriginal Peoples in Canada experience disproportionately higher rates of mortality and morbidity and lower life expectancy compared to non-Aboriginal People in Canada [11–16]. For instance, the First Nations Regional Health Survey found that 63 % of First Nations adults reported living with at least one chronic health condition, with high blood pressure, arthritis, allergies and diabetes ranking most prevalent [17]. Lung cancer incidence rates among Inuit peoples are the world’s highest and rising [18]. Additionally, mental health problems are highly prevalent among Aboriginal Peoples and can be linked to oppression and profound disruption of their traditional lifestyles [19]. This history invites many to reframe Aboriginal Peoples in Canada not as inferior (i.e., as explicitly stated in past Canadian federal policy), but as remarkably resilient given the fact that Aboriginal Peoples and communities are striving and growing across Canada [20].

In addition to Aboriginal Peoples, colonialism also negatively impacts the health of non-European newcomers in Canada [21]. Kirkham [22] notes that certain populations, including immigrants, are disadvantaged by the persistent colonial notion of white superiority in healthcare practices [22]. Furthermore, Canadian-trained healthcare providers (HCPs) involved in global health initiatives are at risk of becoming agents of colonialism in other contexts if they do not develop the skills to critically examine and deconstruct the Western dominance, racism and ethnocentrism underpinning healthcare practice [11].

Postcolonialism is a powerful theoretical approach that enables HCPs to understand how unequal power relations create health inequities [5, 23]. Generally, postcolonialism can be thought of as an interdisciplinary approach to revealing, analyzing and responding to the legacies of imperialism that permeate the healthcare system and create health inequities [21, 24–26]. It has been argued that the prefix ‘post’ implies the end of colonialism; however, we share the view with other scholars that postcolonialism does not dismiss the ongoing realities of colonialism [1], but rather intentionally trains a lens on the emergence of new patterns of inequities [27]. Moreover, postcolonialism can help HCPs understand how they may unwittingly perpetuate colonialism through their practices [11, 28, 29], given that healthcare systems cannot be separated from their broader sociocultural contexts [30].

Despite the acknowledged importance of including postcolonialism within clinical practice and education [5, 11, 28, 31], there is little research investigating how it has been integrated into healthcare training programs. Rare exceptions include the work of Mkandawire-Valhmu & Doering [25] and Mill et al. [32], each of whom discuss the engagement of a postcolonial framework in training for nursing students in the US and Canada, respectively, in preparation for internships abroad [25, 32]. More common has been engagement with the concept of *cultural safety*, which has been viewed as “a vehicle for translating postcolonial concerns into praxis” [33]. Cultural safety was introduced in 1990 by Ramsden [34], and developed throughout the 1990s by Indigenous nurses in New Zealand to address power relations and their impact on health disparities between the Maori people of New Zealand and the descendants of British colonizers [3, 5, 30, 35, 36]. Cultural safety is an approach that encourages HCPs to understand culture as context-dependent and power-laden [28, 30], thereby facilitating the integration of postcolonial theory into healthcare education and practice [3, 5]. Importantly, it is the receiver of care who decides what constitutes culturally safe practices [35].

In this study, we focus on *postcolonialism and health (PCH) in the Canadian context*, which includes: (1) how the legacies of colonialism intersect with the social determinants of health^2^ to produce disparities in health status among populations that Canadian HCPs may encounter in Canada and in ‘global health’ settings, and; (2) how colonialism has shaped the ways that Canadian HCPs and others understand health, illness, healthcare, medicine, well-being and ability.

While the relevance of PCH to healthcare education has been recognized, literature currently lacks guidance on how to incorporate PCH into healthcare training programs. Furthermore, literature in this area largely comprises expert opinion or anecdotal reports as opposed to primary research. Therefore, the purpose of this study was to explore the perspectives of individuals with insight in PCH in the Canadian context regarding content related to PCH that should be included in all Canadian healthcare training programs, and how it should be delivered.

## Methods

This research is inspired by critical theoretical concern with challenging the status quo in healthcare training programs in Canada [37]. However, the inquiry itself is interpretivist in nature, insofar as the researchers co-constructed meaning with the participants about the content and delivery of PCH in Canadian healthcare training programs [38].

### Participants

Participants were eligible for the study if they had insight in PCH in the Canadian context and spoke English fluently. In our sampling criteria, we strove to include perspectives from participants who: (a) had experience teaching postcolonialism in a healthcare training program in Canada; (b) occupied various roles such as clinicians, academics and policy makers, and; (c) worked or taught in the field of disability or rehabilitation. Additionally, voices of Aboriginal People in Canada were specifically sought, given this population’s unique experiences of European colonization in Canada [5, 28]. Purposive and snowball sampling were employed to recruit participants to ensure sampling criteria were satisfied. Participants were invited to participate in the study by email.

This study was conducted in accordance with the Tri-Council Policy Statement (which is the Canadian authority on ethical conduct of research involving humans) with particular attention to Chapter 9: “Research Involving the First Nations, Inuit and Metis Peoples of Canada” [39]. The Director of Research and Policy at the Canadian Aboriginal AIDS Network, who is an Aboriginal woman, co-led this study (RM). Ethics approval was obtained from the University of Toronto, 29557.

### Data collection

In-depth semi-structured interviews were conducted, each lasting 40 to 60 minutes. Interviews were performed by four investigators in person, or by telephone or Skype, based on geographical considerations and participant preference. Participants were sent a Letter of Information detailing the goals of the study and informed consent was obtained verbally from participants. Interviews were digitally recorded and fieldnotes were written throughout and following each interview. Interviews were transcribed verbatim. The interview guide was informed by the research objectives and by relevant literature. Questions were open-ended and explored participants’ experiences teaching or working in this area, and their impressions about content related to PCH that should be included in Canadian healthcare training programs and considerations for how content should be taught. Pilot testing of the interview guide enabled improved flow and phrasing of the questions.

### Data analysis

Data analysis was conducted collaboratively and consisted of six phases as per the DEPICT method [40]. The first step of this process, *dynamic reading*, involved each researcher reading a subset of transcripts and making note of broad concepts that emerged inductively. In the second DEPICT stage, *engaged codebook development*, these concepts were physically mapped to facilitate the emergence of codes derived inductively from the data, which became the draft codebook. The draft codebook was piloted by four of the team members who independently coded the same two transcripts and compared results to ensure stability of the codes. The revised, final codebook was then used to code each transcript twice independently in the *participatory coding* phase to ensure all relevant data were captured. Codes were entered into NVivo software to facilitate data organization. Data in each code were then descriptively analyzed by team members in the fourth DEPICT phase, *inclusive reviewing and summarizing of categories*, resulting in a set of descriptive analyses (one/code). In the fifth phase, *collaborative analyzing,* the research team used a series of meetings to iteratively reflect on how the results contributed to answering our research questions. The final DEPICT phase, *translation,* involved transforming the study findings into a visual schematic and accompanying text [40].

### Rigour

Qualitative research is iterative versus linear and, thus, strategies for verification (“checking, confirming, making sure and being certain” [41]) were built into all steps of the research process and not restricted to post-hoc evaluation strategies [41]. Strategies for enhancing the trustworthiness of the analysis involved *clarifying researcher bias* which included reflexive examination of our past experiences and orientations to be more aware of how our social locations served to shape the interpretation and approach to the study [42]. The DEPICT method of collaborative analysis provided a framework for explicating our analytic steps, while promoting creativity within the process. This approach also allowed for equitable and inclusive relationships among the junior and senior members of our research team [40]. An audit trail, or copy of all documents reflecting methodological changes over time, was maintained for confirmability. Finally, by engaging in *rich, thick description,* we sought to provide sufficient descriptive detail of the participants and the context to assist with transferability of the data [43].

## Results

**Fig. 1:**
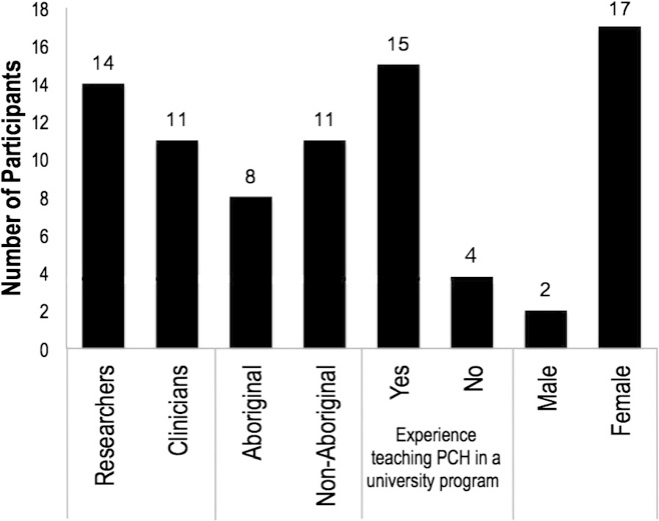
Participant characteristics

**Fig. 2:**
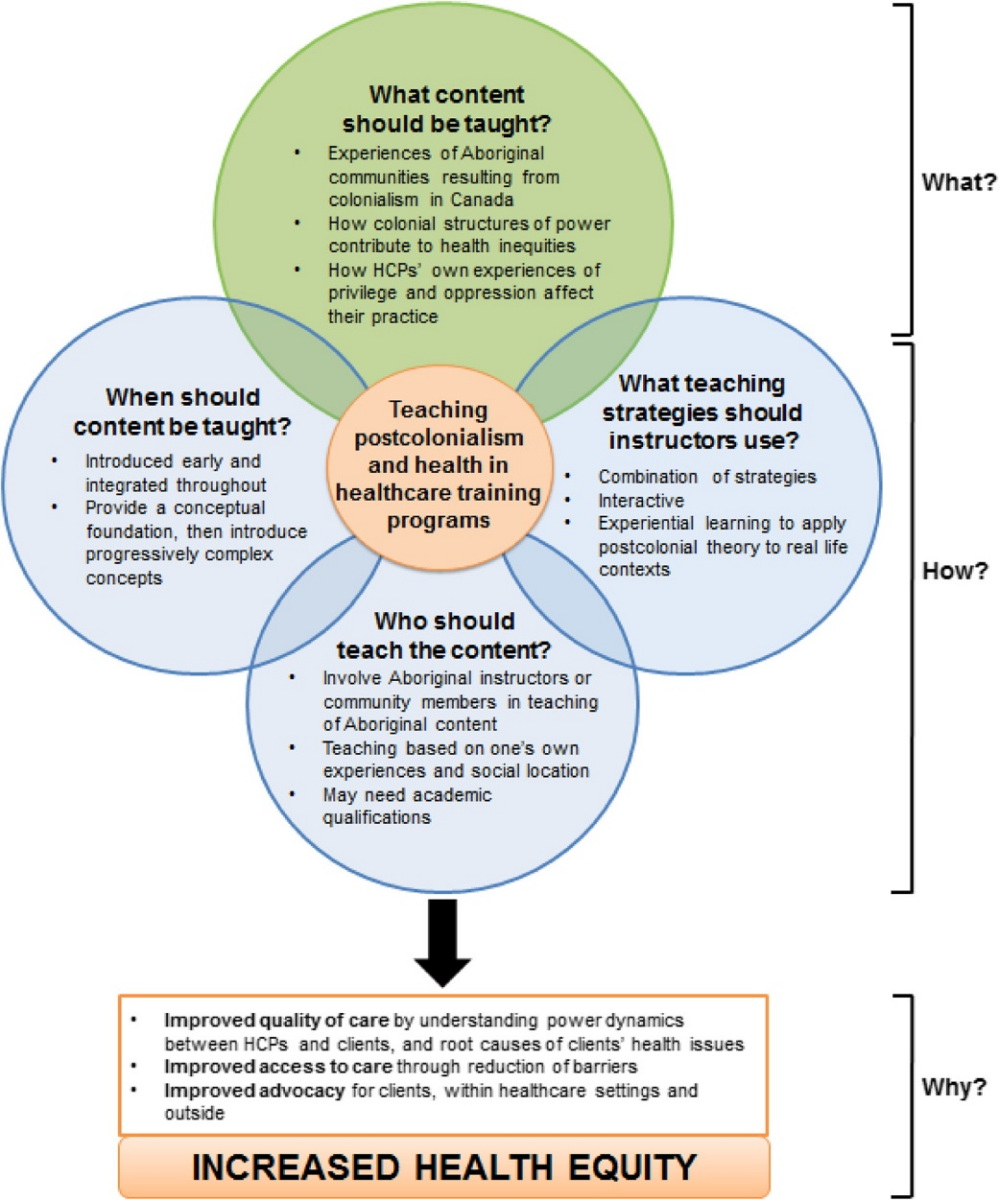
Participants’ perspectives on incorporating postcolonialism and health into Canadian healthcare training programs

A total of 19 participants were interviewed during this study (see Fig. 1). Participants’ perspectives on incorporating PCH into Canadian healthcare training programs were analyzed into three themes: (1) content related to PCH that should be taught; (2) how this content should be delivered, and; (3) why this content should be taught (see Fig. 2). Participants indicated how considerations regarding PCH content influence its delivery, which is depicted by the overlapping circles in Fig. 2. Below, we present these themes in detail.

### What content related to postcolonialism and health should be taught in Canadian healthcare training programs?

Three distinct but complementary content areas emerged based on participants’ perspectives: *the experiences of Aboriginal communities resulting from colonialism in Canada, how structures of power rooted in colonialism continue to create health inequities in Canada* and *how Canadian HCPs’ own experiences of privilege and oppression affect their practice.*

#### What experiences have Aboriginal populations had resulting from colonialism in Canada?

Participants expressed the necessity of teaching about the history of the colonization of Aboriginal Peoples in Canada when discussing Aboriginal health. There was agreement that most students do not enter healthcare training programs with sufficient knowledge in this area, especially as it relates to health and healthcare delivery.

Participants emphasized the importance of understanding how historical events link with an individual’s and community’s present day health status and healthcare experience. Many participants cited the Canadian residential school system as a salient but poorly-understood example of an historical event with a “multigenerational impact” on health status. Residential schools were boarding schools created by the Canadian government in the late 1880s and administered by churches that forcibly separated Aboriginal children from their families, forbade Aboriginal children from acknowledging their culture or speaking their languages, and often involved experiences of abuse [44, 45]. Most residential schools in Canada closed by the 1980s; the last closed in 1996. Participants noted that the residential school experience has led some Aboriginal People to mistrust and fear government institutions, including the healthcare system. One participant explained:

Those who were left behind, and traumatized by the abduction of their children, turned to alcohol to try and dull the pain and the grieving. They were no longer able to understand and communicate with their own children…So as those children grew and started their own families, many of them had turned to alcohol as well because they were foreigners in their own families…So there began the cycle of the impact.

Additionally, the *Indian Act* (1876) and the “Sixties Scoop” were described by participants as important in the education of HCPs. The *Indian Act* gives the Canadian government authority to decide who qualifies as an “Indian” as well as what land is included in First Nation reserves [4, 7]. Some participants indicated that students should learn how the *Indian Act* continues to affect First Nations, Inuit and Métis communities in Canada by determining access to healthcare and funding for health-related issues. Similarly, the “Sixties Scoop” refers to a period in the 1960s that saw the removal of Aboriginal children from their families into child welfare systems, usually without the permission of their families or Bands [46]. Participants emphasized that such trauma continues to negatively influence the health status of people belonging to Aboriginal communities in Canada. However, participants also expressed the importance of acknowledging the strengths and resilience of Aboriginal communities in the face of these challenges, in order to counteract negative stereotypes.

#### How do structures of power rooted in colonialism continue to contribute to health inequities in Canada?

Participants believed students should recognize that colonialism is not over: “You need to understand it is present. It’s present and [Canadians] are totally embedded in it.” In particular, participants explained that understanding why individuals and communities experience health inequities is linked to understanding how systems rooted in colonial practice can influence an individual’s health status:

If you look at the people who are most marginalized, it’s not that they are naturally in this position, it’s because we live in a society that puts them in that position… [I]f we are trying to improve their health we need to understand the systems that are going to continue to undermine their health as soon as they walk out of our office.

Some participants also cited the use of population health statistics as a useful method to explicate health inequities.

Participants agreed that students can better understand the effects of colonial systems of power when using postcolonialism in combination with existing concepts like the social determinants of health:

When you begin to take a look at an infection from a postcolonial lens, they’re not only thinking about bodily health alone. They begin to see that those health outcomes are dependent on social circumstances…And they begin to make those linkages into the sociopolitical context in which healthcare exists, in which these health inequities exist.

Participants also discussed that students should learn how the concepts pertaining to health and medicine are rooted in colonialism and continue to disproportionately marginalize certain populations. Particularly, participants emphasized that HCPs’ views on health and medicine are dominated by a Eurocentric worldview which limits what is accepted as legitimate knowledge: “Not everyone sees the world the way that white, Western Europeans do…we need to realize that there are other ways of looking at health and they are just as valid.” They described how a Western worldview privileges the individualistic, biomedical perspective and consequently devalues other forms of knowledge, such as traditional Aboriginal knowledge. Further, HCPs can unknowingly perpetuate colonial practices by imposing dominant ideas related to health and medicine on the populations they treat. Some participants also emphasized that students should learn about the unique health needs of local populations and understand the diversity within Aboriginal communities in order to avoid generalizing and stereotyping.

#### How do Canadian HCPs’ own experiences of privilege and oppression affect their practice?

Participants emphasized the importance of learning about one’s own social location, particularly positions of power and privilege, as this knowledge is essential to establishing optimal therapeutic relationships between HCPs and clients:

My goal is to try to help nurses enter into practice with an awareness of their own privileges and the ways in which they’re positioned and benefitting from ongoing colonial practices. To help them be critically aware of how judgments and stigma play out. Not just play out in the interpersonal realm, but maintain relations of inequity and how they, in fact, benefit from those.

Related to learning about one’s privilege, participants believed students should reflect on their own assumptions and biases.

Participants identified dealing with the reactions of students, including denial and anger, as a consistent challenge: “These are sensitive issues because you’re deconstructing what [students] have learned throughout their lives…And by unpacking it you’re dissembling their worldview.” Participants explained that it is important for instructors to understand that the experience of learning about one’s privilege is a potentially traumatic experience, one that requires debriefing. One participant explained teaching about PCH first from a structural perspective and then shifting the locus of analysis to an individual’s own privilege may be an effective way to mitigate these reactions. When commenting on the learning needs of HCPs, participants offered reflections that suggested that most (but not all) students in healthcare training programs belonged to socially privileged groups.

Participants also described the notion of becoming “culturally safe” as important for future HCPs. While the terms *cultural safety* and *cultural competency* were often used interchangeably or in combination, some participants distinctly preferred cultural safety. These participants argued that cultural safety incorporates aspects of postcolonial theory into education regarding culture and therefore may be useful to help students understand how postcolonialism applies to healthcare. One participant noted that this approach “speaks more to a form of negotiation that…kind of inverts power relations that we have with healthcare providers and their patients.” Another participant described how the idea of cultural competency is problematic:

Some people, non-Aboriginal People in particular, they’re not sure how to approach the issue of culture in their direct care…It doesn’t mean you learn everything about a single culture, because who can do that? And besides, even people of a particular culture, you learn culture your whole life. You’re born into it, you’re raised in it, you’re learning it probably until the day you die…this notion of competency in someone else’s culture is ridiculous.

### How should content related to postcolonialism and health be taught in Canadian healthcare training programs?

Participants’ perspectives on how to teach PCH content were categorized into three themes: *the teaching strategies that instructors should use, who should teach PCH content to HCPs* and *when this content should be taught.*

#### What teaching strategies should instructors use?

Participants indicated that the delivery of PCH content requires a combination of various methods and depends on factors such as class size and students’ level of postsecondary education.

Participants agreed that this type of content usually works best when it is interactive, whether that involves case studies, small group work or class discussion. Particularly, some participants discussed the importance of evoking an emotional response from students:

I want you to remember the things that you remember because it’s like a train wreck; it’s burned into your memory because it touched you… If it didn’t get to you at that level, then you didn’t truly learn it.

Several participants raised the importance of including the lived experience of Aboriginal People, which may involve inviting guest speakers into the classroom, conducting sharing circles with Elders and using various media (e.g., film, literature, art).

Participants touted experiential learning as an important way to teach PCH content because it encourages students to better understand the individuals that they might work with and see “how differences in power might manifest.” This may include attending local Aboriginal events, participating in traditional cultural practices or ceremony (e.g., sweat lodges), clinical placements, site visits to Aboriginal cultural organizations and visiting Aboriginal communities. However, one participant emphasized that students need to experience the Aboriginal community in a way that is not invasive. Participants also highlighted the necessity for student preparation before engaging in experiential learning, such as learning about the community as well as considering their own social positions and goals for the experience.

Some participants identified resistance from students and faculty members as a potential barrier to delivering this content. Particularly, participants reported that students do not always understand how postcolonialiasm is applicable to their clinical practice and may prefer to spend time learning technical skills. One participant discussed how it is also difficult to convince faculty members about the relevance of this content:

I think where everybody is fighting and struggling for more time to teach students in a limited program, this sometimes is seen as a ‘nice to know’ not a ‘need to know’…sometimes it actually means convincing your colleagues as well that this is really valuable information.

Some participants indicated that students might take content related to PCH more seriously if it were explicitly included in course objectives and tested.

#### Who should teach the content?

Participants’ remarks regarding who should be involved in teaching depended on the specific content being taught, an interdependency that is illustrated in Fig. 2. For example, participants were not prescriptive about who should teach about privilege and power, or “whiteness.” Participants also emphasized that educators should be cautious of teaching lived experiences if they are “outside of that experience.” To that end, participants indicated that Aboriginal educators should be directly involved in the design, review and teaching of curriculum related to Aboriginal history, tradition and present-day issues. They discussed this as an important consideration for building capacity within Aboriginal communities, while also bringing unique expertise into clinical training programs. Participants acknowledged the limited number of individuals from Aboriginal communities who may be available to teach this content (given their other competing demands in addressing Aboriginal inequities), but emphasized that every effort to identify and build relationships with these instructors should be taken.

In particular, there was recurring discussion of forming partnerships with *Elders*, which refers to people who are recognized within Aboriginal communities as possessing special knowledge of history, culture and traditions.

Regardless of the content, most participants agreed that inclusion of instructors who have relevant personal experience is essential but might not be sufficient:

It’s not what experiences you have. It’s how critical you are of them and how much analysis you’ve done of them. So, the experience doesn’t guarantee that you can analyze it.

There was disagreement among participants regarding the academic qualifications that instructors need to teach this content. While most participants felt that higher education and status as a lecturer at a post-secondary institution should not be a requirement, others felt it may be important depending on the nature of the content. Finally, participants believed that in addition to having insight into issues related to PCH, instructors who teach this content should be aware of the importance of creating a safe space for educators and learners.

#### When should this content be taught?

There was consensus that content related to PCH should be introduced early in curricula and integrated throughout in a longitudinal approach. Concepts that students can more readily grasp, such as cultural safety and social determinants of health, should be introduced earlier in the curriculum. These ideas prime students for more complex topics such as the root causes of health disparities, including the colonial processes that continue to disadvantage some and privilege others in Canada.

Participants cautioned against teaching PCH content as a single class or course. Rather, instructors should strive to include concepts relating to PCH into various parts of curricula and revisit critical concepts regularly to emphasize their importance and scaffold learning.

Participants also noted that students should demonstrate their understanding of this material prior to interacting with patients, especially those who are adversely affected by colonization, including Aboriginal and immigrant populations. Many participants also noted that knowledge of this material would facilitate and enhance interactions with *all* patients, regardless of their background because of the insights students gain about *othering*.

### Why should this content be taught?

Ultimately, participants agreed that *it is essential to include content related to PCH in healthcare training programs in order to increase health equity*. Some participants also emphasized the moral and ethical responsibility HCPs have to work toward this goal. Participants discussed several ways in which including this content may help to achieve this goal, as discussed below.

Participants emphasized that learning content related to PCH would improve the quality of care delivered by HCPs to populations negatively impacted by colonialism. HCPs would be more aware of the intricate power relations between themselves and clients and prepared to address this power differential without harming clients. Specifically, in a clinical context, participants highlighted that HCPs would be able to determine the root cause of a client’s health issues, and therefore, deliver more effective and holistic interventions.

In addition, participants discussed how improved quality of care increases access to healthcare services. Participants describe that some individuals, particularly from Aboriginal populations in Canada, do not access health services due to previous negative experiences and mistrust of the healthcare system. One participant explained:

If you experience a health practitioner who seems to understand you, you’re more likely to go see that health practitioner, right? It comes down to the way health practitioners will treat their Aboriginal clients or patients.

Additionally, some participants argued that learning about PCH would equip HCPs with the tools required to become better advocates for their clients both within the healthcare system and society at large. Participants agreed that improved quality of care, access and advocacy are stepping stones to increasing health equity:

[Learning about content related to PCH] would hopefully encourage more Aboriginal People to engage in care because the environment is seen as safe …They become a better provider of care…of course people’s health is going to benefit because they’re accessing care more often…If you’re an advocate for your client, then you’re going to help them to navigate other systems that might not be as culturally safe for them. So I think it has a huge ripple effect.

In summary, participants believed that including PCH content should include a foundational history of colonialism in Canada and how power structures in society operate to create different lived experiences and health inequities. Participants also suggested PCH content should be integrated longitudinally though a variety of interactive teaching strategies and developed in collaboration with local communities in order to increase health equity.

## Discussion

### Postcolonialism is essential in healthcare education and practice

The literature regarding postcolonialism and healthcare education is largely theoretical or anecdotal. This study is the first to empirically examine perspectives of key informants regarding content related to PCH that should be included in Canadian healthcare training programs, and how this content should be delivered. Additionally, participants in our study reaffirmed that content related to PCH should be incorporated into all healthcare training programs in Canada [3, 5, 25, 32, 47]. Results may guide healthcare training programs regarding PCH content to consider for inclusion and how to incorporate it into the curriculum. The findings of this study also call on Canadian regulatory bodies and professional healthcare associations to consider the inclusion of postcolonialism in the standards for clinical practice and accreditation. Finally, the results encourage working with Aboriginal communities to support the participation of Aboriginal People in curriculum development and instruction.

### Beyond cultural competency

This study highlights the need for healthcare educators to critically evaluate how they teach about “culture” and the social determinants of health. *Cultural competency* is an approach focused on equipping HCPs with the knowledge, skills and attitudes to ensure successful interactions with patients of diverse cultural backgrounds [30, 48]. Campinha-Bacote [49] conceptualizes cultural competency as promoting cultural openness, cultural knowledge, cultural awareness and cultural desire, in order to positively shape one’s attitudes regarding difference [49]. However, our findings align with Dogra et al. [50] who have argued that cultural competency often inappropriately presents culture as a fixed set of beliefs, traits and behaviours embodied by all members of a group [50]. Further, Rentmeester [51] noted that the term ‘competency’ infers that clinicians can become experts in the cultures of other peoples [51]. This approach to education in cultural competency has the potential to reproduce stereotypes and inequities, as opposed to dismantling them.

Our findings thus support the call to consider not only cultural competency, but to also encourage HCPs to develop a “critical consciousness” [52] that allows them to examine health and medicine within social, political and historical contexts [52]. *Cultural safety* is well-suited for this purpose given that it has been conceptualized as enabling critical reflection on power relations in healthcare [36]. However, Southwick et al. [53] have criticized cultural safety for “[continuing] to define ethnic minority culture as other than the dominant culture, [which] has the unintended consequence of reinforcing a discourse of binary dialectics” [53]. Therefore, they promote a new model of cultural diversity that reconstructs marginality as a site where complex inter-cultural interactions take place [53]. Similarly, Van Herk et al. [54] present intersectionality as a theory that illuminates how power structures intersect in distinct ways to impact peoples’ health [54]. An intersectionality approach can offer educators an additional lens for helping to make sense of the multiple ways in which colonization operates (e.g., via racism, sexism, heterosexism) to influence health and well-being. Additional guidance may be found in the field of anti-racist pedagogy, which involves deconstructing systems of power to avoid perpetuating racism [55, 56]. Anti-racist pedagogy has been described as enabling the development of a critical consciousness through investigating entrenched systems of oppression and colonial history [48]. A postcolonial lens thus invites healthcare educators to think more deeply about the colonial assumptions embedded within their teaching on topics of “culture”, “race” and health inequities.

### Challenging knowledge and values in current healthcare practice

An emergent theme in our results was that clinicians, educators and students need to challenge ideas and assumptions that they hold as true and “taken for granted”. In particular, our participants explained that HCPs require an awareness of how the Western worldview dominates in healthcare and devalues Aboriginal ways of knowing. Similarly, Holmes et al. [29] explain that colonialism permeates the health sciences and privileges certain kinds of knowledge, such as evidence-based health sciences [29]. Lavallee et al. [12] explain that HCPs need to embrace Indigenous knowledge and teachings in order to help their patients heal [12]. Therefore, our findings support the need to deconstruct notions of truth, evidence and legitimate knowledge in the health sciences, a task that could be well-supported by postcolonial theory [23, 26].

### Wise practices for healthcare education research

Future research should investigate how to effectively deliver content related to PCH to students in healthcare training programs. This is not to suggest the development of *best practices*, which can be impractical and overly reductionist given “that we live in a heterogeneous and changing world” [57]. Rather, the concept of *wise practices* can be a basis from which to engage Aboriginal and other communities in research to investigate this inquiry [58, 59]. Future research should also examine the political, institutional and logistical barriers to implementing PCH into the curricula of healthcare training programs. Finally, while the current study was located in Canada, future research should explore reciprocal lessons for healthcare education in countries such as Australia and New Zealand that share similar histories of colonization, as well as countries in Asia, Africa and Latin America that have been so profoundly shaped by colonial practices.

### Limitations

First, the design of this study is not intended to arrive at results that are generalizable. Rather, the goal of this type of inquiry is to develop better, more nuanced understanding of a complex phenomenon and to identify new knowledge that is *transferable* to other contexts [43]. As such, the findings should not be read as a mandate for all healthcare training programs, but as new ideas that should be considered for their transferability to particular sites.

Second, we did not purposively sample participants with insight into immigrant and refugee health as it relates to postcolonialism. Therefore, the findings regarding PCH content and delivery may not apply to teaching about these populations. Moreover, the paucity of considerations for clinical practice in global health settings may also be attributed to sampling. Another limitation is that the phrase ‘PCH in the Canadian context’ in our interviews may have been interpreted by participants to mean the Aboriginal experience in Canada. This may further account for the lack of recommendations addressing the broader scope of postcolonialism within the Canadian context (i.e. beyond Aboriginal health).

Finally, our results have focused largely on the view of Aboriginal participants and the impact of colonization on Aboriginal Peoples in Canada. We did not explore the voices or experiences of non-Aboriginal People (e.g., from Africa, Asia or the Caribbean) who are also racialized and would have important insights about the impacts (past and present) of colonization on their health. This is an important area for future research.

## Conclusions

Postcolonialism is viewed as a theoretical approach that can enable clinicians and students to understand and respond to the ways that colonialism continues to create and sustain health inequities. This empirical study may provide healthcare educators with guidance regarding PCH content to consider for inclusion in healthcare training programs and how to incorporate it into curricula. In the Canadian context, curricular PCH content should: (1) include a foundation in Canadian colonial history; (2) be integrated longitudinally through a variety of educational techniques, and; (3) be developed in collaboration with local communities. These findings may offer transferable insights regarding clinician training in other countries with similar colonial histories. The results of this study may galvanize healthcare training programs to include PCH content into their curricula and elevate its importance for equitable healthcare provision. Overall, this study joins the chorus of voices calling for critical reflection on the limits and harms of an exclusively Western worldview, and the need for action to name and correct past wrongs in the spirit of reconciliation and justice for all.

## Abbreviations

PCH: Postcolonialism and health
HCPs: Healthcare providers

## Footnotes

1 The term ‘Aboriginal’ includes First Nations, Inuit and Métis peoples [44].

2 The ‘social determinants of health’, which include race, culture, disability, sex and socioeconomic status, strongly influence an individual’s health status, perhaps more so than biomedical and behavioural factors [13, 60].
